# Condom use and risk factors of inconsistent or low use of the condoms during heterosexual anal intercourse in sub-Saharan Africa: a scoping review

**DOI:** 10.4314/ahs.v22i1.3

**Published:** 2022-03

**Authors:** Princess Nyoni, Nigel James

**Affiliations:** 1 International Training and Education Center for Health (I-TECH), 10 Natal Road, Belgravia, Harare; 2 Department of Health Policy and Administration, The Pennsylvania State University, University Park, PA, 16801, USA

**Keywords:** Heterosexual anal intercourse, condom use, sub-Saharan Africa

## Abstract

**Background:**

Anal intercourse (AI) has been reported to be the riskiest among other sexual intercourses in spreading human immunodeficiency virus (HIV) and the risk could be minimized by the use of condoms. Whilst AI is believed to be practised mainly by men who have sex with men, AI has also been reported to occur in heterosexual relationships. However, data on condom use during heterosexual AI are inadequate in sub-Saharan Africa.

**Method:**

A scoping review of English language published articles on condom use during heterosexual anal sex, whose studies were conducted in Sub-Saharan Africa from January 2010 to May 2020 was conducted. Articles were searched systematically on PubMed and Google Scholar electronic databases. Heterosexual AI was defined as penile penetrative anal sex between a man and a woman regardless of the sexual orientation of the 2 parties involved in the act of heterosexual AI.

**Findings:**

A total of 21 studies were eligible for analysis. Most of the studies (17 out of 21) reported females to be involved in heterosexual AI whilst 9 out of 21 studies reported males to be involved in heterosexual AI. The lifetime prevalence estimate of condom use during heterosexual AI ranged from 29%–97.5%. Other prevalence estimates of condom use during heterosexual anal intercourse were reported over various recall periods which were: 12 months' recall period with prevalence estimates ranging from 2.9%–59%; prevalence estimates for the past 3 months which ranged from 50%–94.4%; 1 month's recall period with prevalence estimates ranging from 5%–96% and prevalence estimates for the last intercourse experienced ranging from 1%–55%. Condom use during heterosexual AI was generally low and/or inconsistent among female sex workers (FSWs), men who have sex with men and women (MSMW) and some women in the general population. There were no risk factors identified in the study for the inconsistent or low use of condoms during heterosexual AI.

**Conclusion:**

Evidence from this study suggests condom use during heterosexual AI could be fairly low especially among groups such as FSWs, MSMW and some women in the general population. Risk factors for using condoms inconsistently or using condoms less during heterosexual AI are not clear. Heterosexual anal intercourse and condom use during the AI practice is generally an under-studied subject in Sub-Saharan Africa. Future studies need to explore on heterosexual AI and condom use practices during AI comprehensively so that there can be concrete evidence on the subject which will inform targeted interventions aimed at reducing HIV among heterosexual populations in SSA.

## Introduction

The Acquired immunodeficiency syndrome (AIDS) pandemic is still a burden especially in sub-Saharan Africa (SSA). Existing evidence has shown that globally, in 2018 about 67.5% people living with the Human Immunodeficiency Virus, HIV (virus which causes AIDS) were living in SSA 1. In addition, global AIDS related deaths and number of new HIV infections in 2018 were more than 60% in SSA, about 61% deaths and 64.7% new HIV infections 1. There are numerous ways in which HIV is spread but the main transmission mode is unprotected sexual intercourse either through heterosexual or homosexual relationships^2^,^3^. In Sub-Saharan Africa, most HIV cases are attributable to heterosexual unprotected sex^2^,^4^,^5^. However, consistent and correct use of condoms during sexual intercourse has been reported to be able to abate the transmission of HIV with some studies reporting up to 70%, risk reduction^6^,^7^. A number of studies in SSA have since researched on condom use in heterosexual sexual intercourse. However, most of the studies in SSA seem to focus more on vaginal intercourse when studying heterosexual sex, neglecting heterosexual anal intercourse.

In many parts of the world especially the developing world including SSA, anal intercourse (AI) is believed to be mainly practiced by men who have sex with men. However, anal sex has been reported to be practised too among heterosexual couples which has been corroborated by various studies particularly from the United States of America^8^,^9^,^10^,^11^,^12^. Heterosexual AI is not well studied in SSA yet anal sex has been identified to carry the highest risk of transmitting HIV. The odds of spreading HIV via unprotected anal sex was reported to be at least 10 times more compared to unprotected vaginal sex^13^ and the Centre for Disease Control also reported unprotected anal sex to spread HIV much more than unprotected vaginal and other types of sexual intercourse 14. Evidence on condom use during heterosexual AI in SSA is not clear and such a deficit presents a missed opportunity in HIV/AIDS response. Therefore, due to the scant synthesise of condom use during heterosexual anal intercourse in SSA, it was worthwhile to conduct a scoping review on the practice.

This scoping review's main aim was to synthesize current evidence on condom use during heterosexual anal intercourse in SSA. Scoping reviews map present literature to a certain research topic, examining the research activity in a topic area and identifying gaps in the existing literature 15. A scoping review can also be of use if the topic has not been reviewed widely or when unclear^16^. Specifically, this review answers the following questions: i) What is the estimated prevalence of condom use during heterosexual anal intercourse in SSA? ii) What are the patterns of condom use during heterosexual anal intercourse in SSA and iii) What are the risk factors of inconsistent or low condom use during heterosexual anal intercourse in SSA? Collated information in this review will document information on condom use practices during heterosexual AI coherently. Documenting information on condom use practices during heterosexual AI will enable identification of gaps in literature to inform future research. The documented information can also provide adjunct evidence which can aid in the implementation of the HIV programmes in SSA.

## Methods

**Figure 1:**
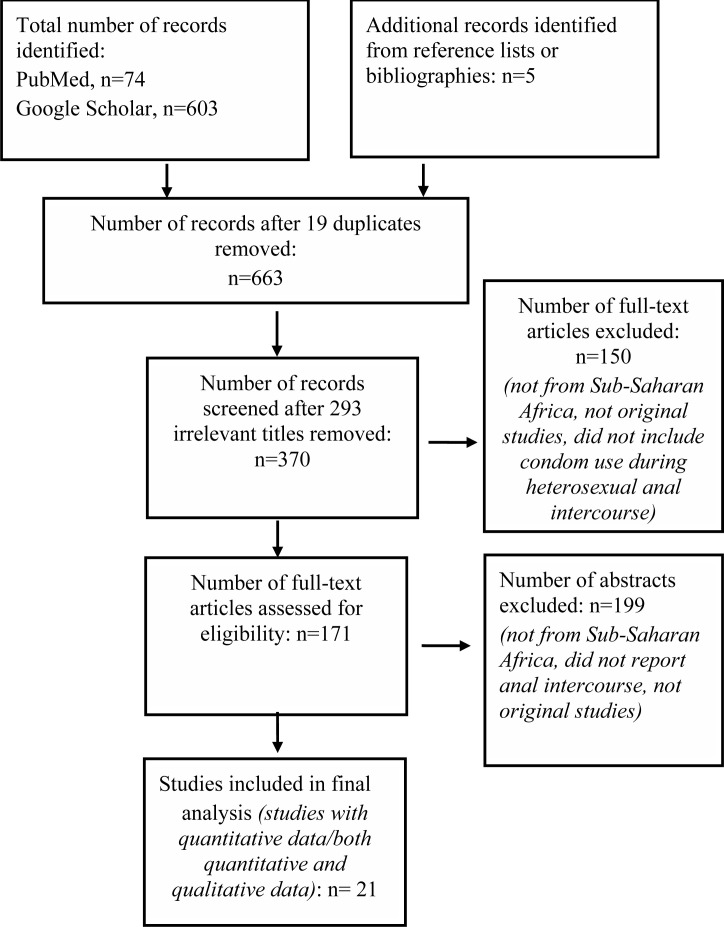
PRISMA flow diagram for the scoping review process

The scoping review was undertaken following the Preferred Reporting Items for Systematic Reviews and Meta-Analyses (PRISMA) guidelines. Fig 1 below shows the PRISMA chart with step by step process followed in this scoping review exercise.

### Search Strategy

Articles were searched on PubMed and on Google Scholar databases. English language articles published from January 2010 to May 2020 were searched on PubMed using the search terms: heterosexual OR women OR female AND anal AND (Sex OR Sexual) AND behaviour AND condom use AND Africa as the Medical Subject Headings (MeSH) terms. English language articles published from January 2010 to May 2020 were further searched on Google Scholar using the key words: heterosexual, women, anal sex behaviour, condom use, Africa with the exact phrase ‘sub-Saharan African countries’. A list of all the identified references was made in Zotero (a reference managing software) and duplicates were removed. Titles were first screened and those which were clearly irrelevant were discarded and abstracts were then screened. Full text articles were opened if the abstract had reported AI and if study was done in SSA or if study area was not specified. Full text articles were also opened from the abstracts whose study areas were not specified in case the studies were done in SSA. Full text articles were screened for condom use during heterosexual AI in sub-Saharan Africa. Eligible articles' bibliographies were screened for any additional relevant articles.

### Inclusion and exclusion criteria

Studies were included if: they were published in English language from January 2010 to May 2020, they were original studies, they were conducted in sub-Saharan Africa, they reported condom use during heterosexual AI and if the studies were based on quantitative or both quantitative and qualitative data. The studies had to contain at least a quantitative section so that the research questions on estimating prevalence of condom use during heterosexual AI and identifying risk factors for inconsistent condom use during heterosexual AI could be answered.

Studies were excluded if: they were not original studies, they reported heterosexual AI but did not report condom use during heterosexual AI, if they reported both homosexual and heterosexual AI in a way that the 2 were indistinguishable, if they were conducted solely or partly outside SSA and if they did not report quantitative data. Heterosexual anal intercourse was defined as the penile-anal penetrative intercourse between a man and a woman regardless of the sexual orientation of the 2 parties involved in the heterosexual AI.

## Results

A total of 21 articles were included in the final analysis. Most of the studies were from South Africa (9 studies), followed by Kenya (4 studies), Nigeria (2 studies) and single studies each from Tanzania, Cote d'ivoire, Senegal, Eswatini and Mozambique. One additional study was conducted in 3 countries; South Africa, Zimbabwe and Uganda. The common study design was the cross sectional study (12 articles), followed by cohort studies (4 articles), clinical trials (2 articles) and single studies each based on observation, in-depth interviews and mixed methods. Over 50% of the studies were based on convenience sampling. All the studies' responses on sexual behaviour were self-reported by participants. The common data collecting technique was the questionnaire; 9 interviewer administered, 6 self-administered and 2 which were not specified how they were administered. The studies' sample sizes ranged from 83 to 4965 though the sample sizes of those who practiced heterosexual AI differed by study. Only 1 study of the 21 focused exclusively on heterosexual AI. Table 1 presents summary of the main findings.

**Table 1:** Condom use during heterosexual anal intercourse in SSA

Authors and year	Study design/ data collecting tool	Participants	Setting	Condom use practices during heterosexual anal intercourse
**Kalichman et al.** **(2010)**^17^	Cross sectional Study/ self- administered questionnaire.	2593 men and 1818 women, (median age 30 years).	Township communities and STI clinics in Cape Town, South Africa.	75% men of the 14% and 50% women of the 10% who reported anal sex used condoms in the past 3 months. An average of 61.96% participants from township communities and 59.99% from STI clinics used condoms during AI in the past 3 months.

**Priddy et al.(2011)** 18	Prospective cohort study/ interviewer administered questionnaire.	200 women who reported exchanging sex for money/presents, (mean age: 28 years, range 18– 55years).	Mukuru neighbourhood, Nairobi, Kenya.	Overall, 37% reported AI and condom use varied with partner types below: *Primary partner* 9.4% sometimes/ always have AI with primary partner and 29% sometimes/ always used condoms. *Regular partner* 35.4% sometimes/always have AI with regular partner and 73.2% sometimes/always used condoms. *Casual partner* 28.7% sometimes/always had AI with casual partner and 73.2% sometimes/always used condoms.

**Van Loggerenberg et** **al.** (2012)^19^	Prospective observational cohort study/interviewer administered questionnaire.	245 HIV high risk women, (mean age: 34.2 years, SD ±10.5).	Durban, South Africa.	11.3% practiced AI once/less per month, 21.9% twice/more and 50% used condoms at last AI.

**Kalichman et al.** **(2011)**^20^	Cross sectional study/self- administered questionnaire.	4965 (3372 men,1593 women) shebeen patrons.	Shebeens in a township 20km away from Cape Town CBD, South Africa.	15% men and 11% women reported AI and 8% of the men and 7% of the women did not use condoms during the AI in the past month.

**McLellan-Lemal et**	Observational study/audio	463 women aged 18–34	150 km within Kisumu	7.3% reported AI in the past 12 months and 97.1% of these did
**Shayo et al.** **(2017)**^22^	**Cross sectional study/** **interviewer administered** **questionnaire and FGDs.**	**356 males and 547** **females, (mean age:** **33 years±12.5 range 15–** **84 years).**	**4 districts in Tanzania.**	**26.4% reported to practice AI and 36.4% always used** **condoms during AI whilst 25% used them during the last** **AI.**

**Maheu-Giroux et al.** **(2018)**^23^	**Cross sectional RDS** **survey/ interviewer** **administered** **questionnaire.**	**466 FSWs, (mean age 27** **years range, 18–57years).**	**Abidjan,** **Cote d'Ivoire.**	**Overall 20% reported to practice AI in a normal week, 24%** **in the past month and 24% in the past year.** **59% used condoms always/frequently during AI in the past** **year and 28% never used condoms.** **Condom breakage during AI reported by 38% in the past** **month.** **Condom use by client type:** ***New clients*** **13% reported AI and 47% used condoms consistently for** **AI.** ***Regular clients*** **13% reported AI and 36% used condoms consistently for** **AI.** ***Non-paying partners*** **14% reported AI and 5% used condoms consistently on AI.**

**Duby et al.** **(2016)**^24^	Multi-site qualitative ancillary study/In-depth interviews.	88 women, (mean age 28.6 years range, 20–40 years).	Durban, South Africa, Kampala, Uganda and Chitungwiza, Zimbabwe.	23% reported AI and 55% of those used condoms. Condom use by country; *South Africa* 13% reported AI and 72.7% of those used condoms. *Uganda* 27% reported AI and 33.3% of those used condoms. *Zimbabwe* 3% reported AI and 33.3% of those used condoms.

**Larmarange et al.** **(2010)**^25^	Survey/self-administered questionnaire.	501 MSMW, 80% under 30 years.	Dakar, Mbou/Thies Saint-Louis, Senegal	1.1% practised AI on last intercourse and 75% of it unprotected.

**Gaffoor et al.** **(2013)**^26^	Double blinded placebo controlled trial/self- administered questionnaire.	1485 women	Durban, South Africa	5.6% reported unprotected AI in the past 3 months.

**Dubbink et al.** **(2016)**^27^	**Cross sectional study/** **interviewer administered** **questionnaire.**	**310 Sotho and 260** **Shangaan women,** **(median age: 30 years** **range 18–49 years).**	**Mopani District,** **Limpopo, South Africa.**	**5.4% Sotho and 4.2% Shangaan women practiced AI and** **overall, 12% reported condom use during last sex act.**

**Cain et al. (2012)** 28	Cross sectional survey/ self- administered questionnaire.	981 men and 492 women, 18 years+, median age 30 years.	Township 20km away from Cape Town CBD, South Africa.	Condom use during AI in past month by whether shebeen patron/not: *Shebeen patrons* 8% women practiced unprotected AI. *Non-shebeen patrons* 4% women practiced unprotected AI.

**Gray et al. (2013)** 29	Single blinded placebo controlled trial/interviewer administered questionnaire.	441 men and 360 women recruited at baseline, aged 18–35years.	Cape Town, Soweto, MEDUNSA, KOSH and eThekwini in South Africa.	*AI among men was not specified as purely heterosexual. 2.8% practiced unprotected AI in Soweto, 2% in Cape Town, 2.6% in KOSH, 4.2% in eThekwini and none practiced AI in MEDUNSA. 2.5% practiced unprotected AI overall.

**Smith et al.** **(2010)**^30^	Cohort study/Prospective, sexual behaviour self- completed diaries.	83 MSMW, aged 18+ years.	Mombasa, Kenya.	12% reported AI with women and 46% of the AI unprotected.

**Ochonye et al.** **(2019)**^31^	Cross sectional survey/interviewer administered questionnaire.	488 FSWs, MSM and PWID, aged 18+ years.	Enugu, Nassarawa, Benue and Akwa-Ibom in Nigeria.	59% FSWs reported AI and 34.6% always used condoms during AI in past 12 months.

**Ybarra et al.** **(2018)**^32^	Cross sectional survey/self-administered questionnaire.	349 males and 588 females, age range 16–24 years.	Secondary schools, Langa, Cape Town, South Africa.	10.88% of females practiced AI and condom use during AI half of the time or less was 28.13% and for more than half of the time 71.88%. *AI among males not specified as purely heterosexual.

**Folayan et al.** **(2015)**^33^	Cross sectional survey/interviewer administered questionnaire.	344 females and 428 males.	Urban and rural townships in Nigeria.	1.9% ever practised AI whilst1% practised AI in past 12 months and condom use at last AI was 1%.

**Musyoki et al.** **(2018)**^34^	**Cohort study/Polling booth** **surveys.**	**3448 FSWs,1308 MSM** **and 690 PWID in 2014.** **2228 FSWs, 1254 MSM** **&598 PWID in 2015.**	**Numerous locations,** **Kenya.**	**12% FSWs reported AI in the past month in 2015 and** **condom use during last AI was 58% in 2014 and 55% in** **2015.**

**Owen et al.** **(2020)**^35^	Cross sectional survey/interviewer administered questionnaire.	325 FSWs, (mean age 26 years, range 16–49 years).	Eswatini	Overall, 44% practiced AI in the past month and 34% used condoms inconsistently. Condom use by client type: *New clients* 37% reported AI, 54% reported inconsistent condom use and 17% reported broken/slipped condom. *Regular clients* 39% reported AI, 67% reported inconsistent condom use and 28% reported broken/slipped condom. *Non-paying clients* 36% reported AI, 76% reported inconsistent condom use and 39% reported broken/slipped condom.

**Lane et al. (2011)** 36	Cross sectional survey/questionnaire (administering method not specified).	363 MSM, (median age 23 years range 18–48 years).	Soweto, South Africa.	16.3% reported any AI with females and 8.8% unprotected AI.

**Sathane et al.** **(2016)**^37^	Cross sectional survey/questionnaire (administering method not specified).	MSMO and MSMW recruited. 496 men in Maputo, 583 in Beira and 353 in Nampula-Nacala.	Maputo, Beira and Nmpula-Nacala in Mozambique.	Condom use practises reported by place; *Maputo* 37.4% had any AI and 15.5% had unprotected AI. *Beira* 24.9% had any AI and 8.2% had unprotected AI. *Nampula-Nacala* 37.6% had any AI and 19.6% had unprotected AI.

Abbreviations: AI-anal intercourse; CBD-Central Business District; FGDs-focus group discussions; RDS-respondent driven sampling; FSWs-female sex workers; MSMW-men who have sex with men and women; MSM-men who have sex with men; PWID-people who inject drugs; MSMO-men who have sex with men only.

NB: The prevalence of anal intercourse was reported first before the prevalence of condom use during anal intercourse to show a clearer picture of what proportion of those practising AI use condoms. However, some of the studies did not report the prevalence of anal sex hence the prevalence of condom use was the only one reported.

### Heterosexual anal sex background and its relationship with human immunodeficiency virus

The prevalence estimates for heterosexual AI ranged from 1.1% among 501 men who have sex with men and women (MSMW)^25^ to 59% among 488 female sex workers (FSWs)^31^. Eight studies reported HIV prevalence and or incidences of participants^17^,^20^,^21^,^23^,^27^,^29^,^35^,^36^. The participants' HIV status and their relationship with AI practices was however noticed in half of the 8 studies^17^,^20^,^35^,^36^. There was no difference in HIV prevalence in relation to AI practices in 2 studies^17^,^35^. In one study conducted in South Africa, the prevalence of HIV was higher in participants who engaged in AI compared to those who did not, with the HIV prevalence much higher in female participants^20^. In another study conducted in Suth Africa again, practising AI was significantly associated with HIV positive status^36^.

### Prevalence estimates of condom use during heterosexual anal intercourse in SSA

Prevalence estimates of condom use during heterosexual anal sex were reported disaggregated by recall periods. Apart from the lifetime recall period, a total of 4 recall periods were identified from the various studies analysed. The four recall periods include the 12 months' recall period, 3 months' recall period, 1 month's recall period and the last intercourse experienced. Study groups and their area were also specified on the prevalence estimates since the studies were focusing on various groups and from various places.

The lifetime prevalence estimate for condom use during heterosexual AI ranged from 29%, among female sex workers (FSWs) in Kenya18 to 97.5%, among men and women in South Africa^29^. For the recall period of 12 months, the prevalence estimate for condom use during heterosexual AI ranged from 2.9%, among women in Kenya^21^ to 59%, among FSWs in Cote d'ivoire^23^. For the past 3 months, the prevalence estimate for condom use during heterosexual AI ranged from 50% 17 to 94.4% 26 among women in South Africa. For the 1 month's recall period, the prevalence estimate for condom use during AI ranged from 5%, among FSWs in Cote d'ivoire^23^ to 96% among men and women in South Africa^28^. The range of using condoms during heterosexual AI reported for the last intercourse experienced was from 1%, among males and females in Nigeria33 to 55%, among FSWs in Kenya^34^.

### Condom use patterns during heterosexual anal intercourse in SSA

Condom use during heterosexual AI was generally low among FSWs and among some men who have sex with men and women (MSMW) populations with prevalence estimates ranging from about 3% to about 70%^18^,^19^,^21^,^23^,^24^,^25^,^27^,^30^,^31^,^32^,^34^,^35^ compared to studies reporting heterosexual AI among men and women in the general population whose estimates were over 90% 20,26,28,29. Studies focusing on men and women in the general population showed that men generally used condoms during AI more than women^17^,^20^. In addition to low condom use, FSWs were also reported to use condoms inconsistently^18^,^23^,^31^,^35^. Condom use during heterosexual AI among FSWs was low with their primary partners/non-paying partners^18^,^23^,^35^. Condom breakage during heterosexual AI was also reported among FSWs and the rate at which the condoms broke ranged from 17% to 39%^23^,^35^. The condom breakage among FSWs was high with their primary/non-paying partners, followed by regular partners and lowest with casual partners/new clients^35^. Lower condom use during heterosexual AI was observed in studies among general men and women populations conducted outside South Africa, (range: 1%–36.4%) 21,22,24,33 compared to the studies among general men and women populations conducted in South Africa, (range:71.9%–97.5%)^17^,^20^,^24^,^26^,^28^,^29^,^32^.

### Risk factors of inconsistent or low use of condoms during heterosexual anal intercourse in SSA

All the 21 studies did not report any risk factors of inconsistent condom use during heterosexual AI. However, one study from Tanzania identified the following reasons for not using condoms consistently during AI from focus group discussions and these include; to avoid reducing pleasure during the intercourse, to prove loyalty to one's partner, unavailability of condoms nearby, inconvenience of condoms during AI (that is anus too dry to use condoms) and some participants perceived AI to be less risky in transmitting HIV^22^. Knowledge that AI conveys highest risk of transmitting HIV was also reported by very few participants, only 6%–10% in other studies^23^,^35^.

## Discussion

Our findings identified the prevalence estimates for condom use during heterosexual AI in SSA to vary but mostly low. Over 60% of the reviewed studies' various prevalence estimates for condom use during AI ranged from about 3% to about 70% with only 2 articles in that range reporting estimates of about 70% and the rest below 60%. Populations such as FSWs, MSMW and women in general are known to be HIV high risk groups^38^ and our study further confirmed that the groups are at high risk of acquiring HIV since majority of the studies identified low prevalence estimates for condom use or inconsistent condom use during AI among these groups. Condom breakage was further reported among FSWs and this could be due to not using lubricants or due to the use of lubricants not suitable for condoms during heterosexual AI as similarly reported in other studies 39,40,41.

It is not clear why condom use during heterosexual AI was identified to be lower among men and women in general populations in studies conducted outside South Africa than in studies conducted in South Africa. However, condom use during heterosexual AI might be low in some countries other than South Africa possibly due to cultural variations which either encourage or discourage condom use as also identified in another study that social norms might either encourage or discourage condom use^42^. Differences in conom use between population groups could also be due to differences in access to condoms as some participants of one study in Tanzania reported condoms not to be easily accessible^22^. Our study is not enough to ascertain the condom use differences trend though. Therefore, further researches could find out the probable reasons for the differences in condom use if available, between nations in sub-Saharan countries.

None of the reviewed studies identified risk factors for inconsistent or low condom use during heterosexual AI. However, some risk factors identified for heterosexual AI could be indirect risk factors of inconsistent or low condom use during heterosexual AI for example experiencing sexual violence/coercion^32^,^35^. Sexual violence perpetrators would most likely not use condoms on their victims hence experiencing sexual violence/coercion could predispose one to unprotected intercourse. Some reasons cited for inconsistent or low condom use during heterosexual AI in this study suggest lack of access to condoms, lack of access or knowledge on condom compatible lubricants for AI and lack of knowledge about AI and its risk in transmitting HIV could be risk factors for inconsistent or low condom use during heterosexual AI.

Evidence from our study shows that anal sex is being practiced during heterosexual intercourse in SSA though its prevalence is not clear. However, extensive studies on heterosexual AI in SSA are deficient including studies on condom use during heterosexual AI in SSA. Only 1 article of the 21 reviewed studies conducted a study exclusively on heterosexual AI. It is therefore imperative for future studies to explore on heterosexual anal intercourse and condom use during the anal intercourse comprehensively, especially given that AI is the riskiest in transmitting HIV. Concrete evidence on heterosexual AI and condom use during the AI practice can provide crucial information which will inform appropriate and targeted interventions aimed at averting HIV/AIDS in SSA.

## Limitations and strengths of the study

Our review only included articles based on published studies. Studies from earlier than 2010 were excluded because we wanted more recent data on the practice. Also, various recall periods and heterogeneity of study populations made it harder to compare prevalence estimates. Most studies used interviewer administered questionnaires to collect data which are less private and this might have led to social-desirability bias such as underreporting AI practises. Most of the studies employed convenience sampling and some recall periods were long which could have led to recall bias. As a result of the above limitations, our study's findings can only be used as preliminary evidence on heterosexual AI practice. Our study however, managed to identify some gaps for instance we identified that data on the risk factors of inconsistent or low condom use during heterosexual AI are deficient and that there is a general lack of studies on condom use during heterosexual AI in SSA. Some of the limitations mentioned above such as use of interviewer administered questionnaires and using convenient samples can also be treated as gaps in the heterosexual AI research which should be addressed by future researchers.

## Conclusion and Recommendations

Evidence from this study suggests condom use during heterosexual AI could be fairly low and the most vulnerable groups for inconsistent or low condom use during heterosexual AI being FSWs, MSMW and some women from the general population. Risk factors for inconsistent or low condom use during heterosexual AI are not explicit. Since most studies employed convenience samples, used interviewer administered questionnaires to collect data which are less private and some recall periods in the studies were long, we therefore recommend;

- Future studies on heterosexual AI to use self-administered tools if possible to collect data so as to minimize desirability bias given the sensitive nature of the AI subject.
- Future studies on heterosexual AI to try and sample participants randomly so that the samples can be representative of the target population and hence the findings can be generalized to the target population.
- To minimize recall bias, researchers in future could avoid asking participants to recall their sexual behaviours which would have happened a long time ago. Generally, more studies need to focus on exploring heterosexual AI and condom use during the practice in SSA so that there can be concrete evidence on the practice which will inform targeted interventions aimed at reducing HIV among heterosexual populations in SSA.
